# New ^β^*N*-octadecanoyl-5-hydroxytryptamide: antinociceptive effect and possible mechanism of action in mice

**DOI:** 10.1038/s41598-018-28355-4

**Published:** 2018-07-03

**Authors:** Thais Biondino Sardella Giorno, Iris Gonçalvez da Silva Moreira, Claudia Moraes Rezende, Patricia Dias Fernandes

**Affiliations:** 1 Universidade Federal do Rio de Janfeiro, Instituto de Ciências Biomédicas, Laboratório de Farmacologia da Dor e da Inflamação, Rio de Janeiro, Brazil; 2 Universidade Federal do Rio de Janeiro, Instituto de Química, Laboratório de Aromas, Rio de Janeiro, Brazil

## Abstract

The present study examined the potential antinociceptive activity of C18 5-HT (^β^*N*-octadecanoyl-5-hydroxytryptamide) using chemical and thermal nociception models in mice. Orally administered C18 5-HT (0.1, 1 and 10 mg/kg) produced significant dose-dependent antinociceptive effects in formalin-, capsaicin- and glutamate-induced licking models. This compound also induced a significant increase in the response to thermal stimuli in the hot plate test, and its antinociceptive effect was not related to muscle relaxant or sedative actions. In a thermal hyperalgesia model, C18 5-HT presented an anti-hyperalgesic profile as evidenced by the increase in the response time of the animals. Furthermore, intraperitoneal (i.p) pretreatment with naloxone (a non-selective opioid receptor antagonist, 1 mg/kg), ondansetron (serotoninergic receptor antagonist (5-HT3 subtype), 0.5 mg/kg) or AM241 (CB1 cannabinoid receptor antagonist, 1 mg/kg) reversed the antinociceptive effects of C18 5-HT in the hot plate model. In the formalin-induced licking model, pretreatment with naloxone reversed the antinociceptive effects of C18 5-HT, as demonstrated by an increase in the paw licking response when compared with the C18 5-HT-treated group. These findings suggest that C18 5-HT has peripheral and central antinociceptive effects and that its mechanism of action involves, ate least in part, opioid, serotoninergic and cannabinoid pathways.

## Introduction

Pain is a noxious sensation resulting from tissue injury and acts as a beneficial response necessary for the preservation of tissue integrity^1^. Clinical treatment of pain currently involves the use of opioid and non-opioid agents. However, both types of drugs present safety profiles with limited effectiveness and numerous side effects. Non-steroidal anti-inflammatory drugs are associated with severe gastrointestinal, renal or liver damage. To date, opioids are the most potent and effective analgesics for pain treatment. However, their severe side effects, such as tolerance and addiction, limit their use^2^. Therefore, the search for new analgesics with higher efficacies and fewer perceived effects is a continuous objective for pain treatment.

During the last few years novel derivatives of 5-HT were described and presented interesting antinociceptive and anti-inflammatory properties^3^. For instance, C18 5-HT (^*β*^*N*-octadecanoyl-5-hydroxytryptamide) was found in the surface wax of green coffee beans^4^ and was synthesized by combining an octadecanoyl unit with serotonin^5^. Some amides in the serotonin class have also demonstrated an anti-inflammatory effect by inhibiting the expression of caspases, a class of enzymes involved in the inflammatory process^6^. Furthermore, Ortar *et al*.^7^ described that another serotonin amide, N-arachidonoyl 5-HT, has agonist activities towards CB1 and CB2 cannabinoid receptor type, as well as being an antagonist of TRPV receptors, confirming its analgesic effect.

The purpose of the present study was to investigate the antinociceptive effects of C18 5-HT using selected chemical (formalin-, capsaicin- and glutamate-induced licking response) and thermal model of nociception in mice. In addiction, the mechanisms of action of this compound was investigated to confirm its antinociceptive effect and use it as a potential antinociceptive therapeutic agent.

## Results

### Evaluation of the antinociceptive effects of C18 5-HT in the formalin-induced licking model

The animals that were orally treated with the vehicle spent 50.5 ± 1.4 seconds and 214.3 ± 3.1 seconds licking their paws in the 1^stt^ and 2^nd^ phases, respectively. Acetylsalicylic acid (ASA, 200 mg/kg) and morphine (2.5 mg/kg) were used as reference drugs. The animals that were orally pretreated with ASA 60 minutes before formalin injection spent 46.8 ± 3.5 seconds and 122.4 ± 11.4 seconds licking their paws in the 1^st^ and 2^nd^ phases, respectively, which demonstrated 42.8% inhibition in the second phase. In the animals that were orally pretreated with morphine, the time spent licking in the 1^st^ and 2^nd^ phases was 30.0 ± 3.1 seconds and 138.4 ± 7.5 seconds, respectively, showing a consequent inhibition of 40.6% and 35.4% in the 1^st^ and 2^nd^ phases, respectively. We also evaluated if a direct injection of 5-HT C18 in mice paw could induce any response. We observed that 5-HT C18 did not demonstrate any nociceptive or behavior response when intraplantarlly injected (data not shown).

**Figure 1:**
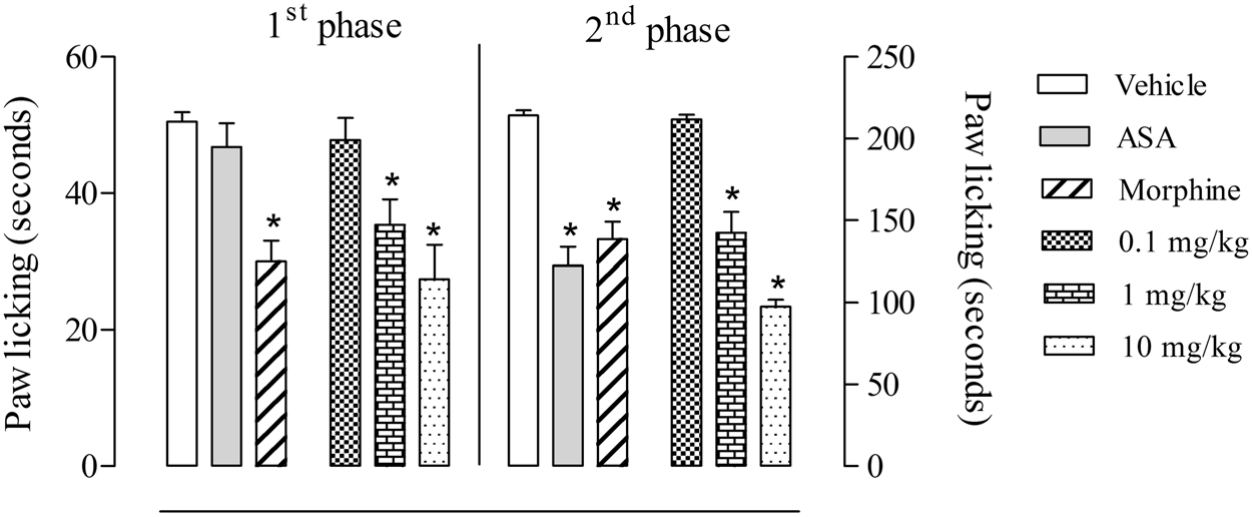
Effect of C18 5-HT on formalin-induced licking response in mice. Animals were orally pretreated with C18 5-HT (0.1, 1 or 10 mg/kg), acetylsalicylic acid (ASA, 200 mg/kg), morphine (2.5 mg/kg) or vehicle (Tween 80) 60 minutes before intraplantar injection of formalin (2.5%). The results are expressed as the mean ± S.D. (n = 6–8) of the time the animals spent licking the formalin-injected paw. Statistical significance was calculated by two-way ANOVA followed by Bonferroni’s test. *Indicates p < 0.05 when compared to the vehicle-treated mice.

Figure 1 shows that C18 5-HT significantly inhibited formalin-induced licking responses in the 1st and 2nd phases at the higher doses (1 and 10 mg/kg), with a 30.1% and 45.7% reduction in the 1^st^ phase and a 33.5% and 54.5% reduction in the 2^nd^ phase, respectively.

### Evaluation of the antinociceptive effects of C18 5-HT in the capsaicin- and glutamate-induced licking models

**Figure 2:**
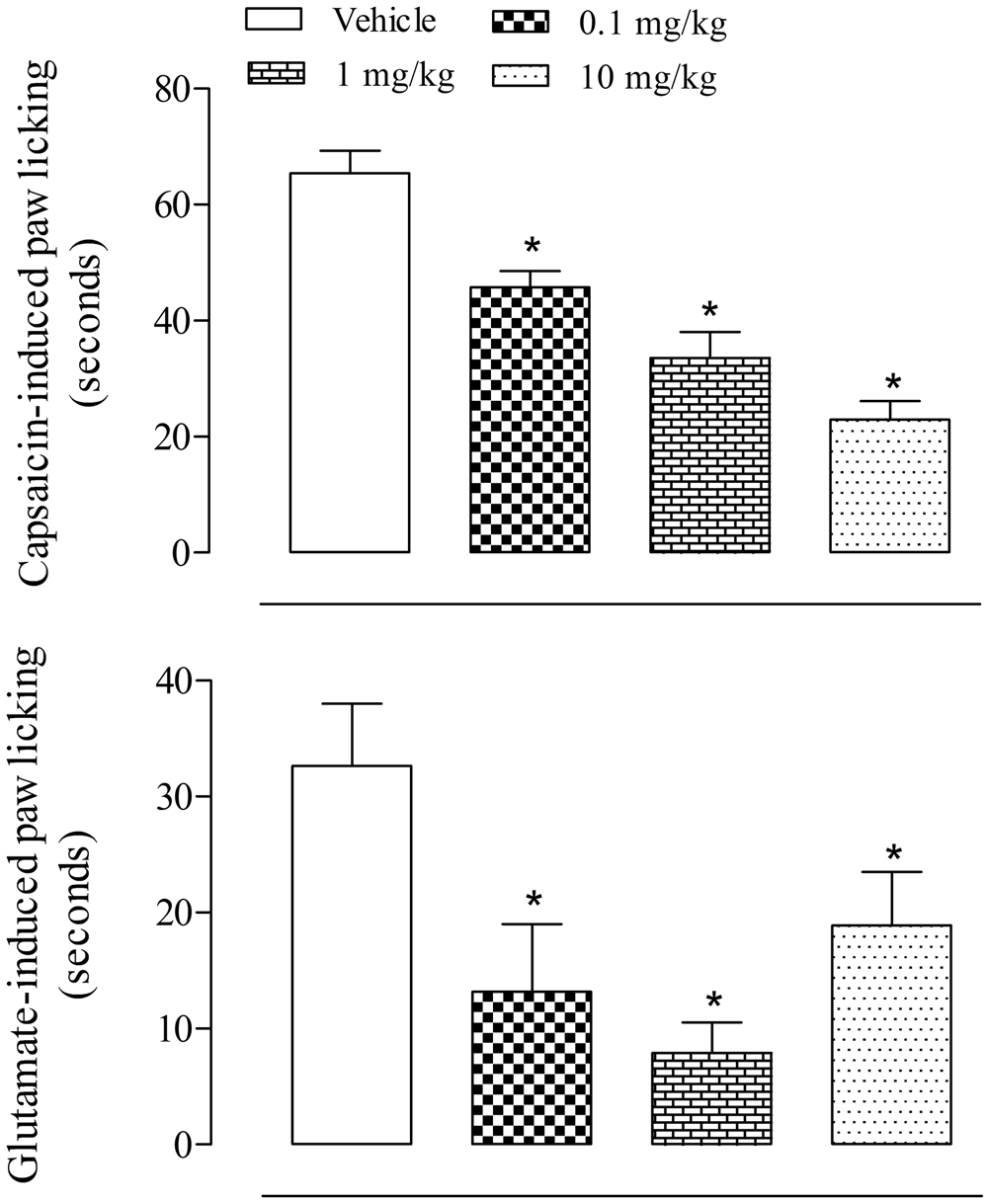
Effect of C18 5-HT on capsaicin- or glutamate-induced licking response in mice. Animals were orally pretreated with C18 5-HT (0.1, 1 or 10 mg/kg), vehicle (Tween 80) or capsazepine (3.8 pg/paw) 60 minutes before the injection of capsaicin (in A, 1.6 μg/paw) or glutamate (in B, 3.7 ng/paw). The results are expressed as the mean ± S.D. (n = 6–8) of the time the animals spent licking the capsaicin- or glutamate-injected paw. Statistical significance was calculated by two-way ANOVA followed by Bonferroni’s test. *Indicates p < 0.05 when compared to the vehicle-treated mice.

Intraplantar injection of capsaicin induces an acute nociceptive response through TRPV-1 receptor activation that is expressed in peripheral sensory neurons. Figure 2A shows that pretreatment with C18 5-HT at the 3 tested doses led to a significant and dose-dependent reduction in licking time compared to the vehicle-treated group (a 30.1%, 48.7% and 65% reduction).

To investigate if the participation of glutamatergic system was involved in C18 5-HT antinociception, we evaluated whether pretreatment with this substance would reverse the antinociceptive effects associated with the intraplantar glutamate injection. Pretreatment with C18 5-HT caused a significant reduction in paw licking induced by glutamate (0.1 mg/kg = 11.6 ± 5.5 seconds (64.7% inhibition); 1 mg/kg = 7.9 ± 2.6 seconds (76% inhibition) and 10 mg/kg = 18.9 ± 4.6 seconds (42.5%)) (Fig. 2B).

### Evaluation of the central antinociceptive

#### effects of C18 5-HT

**Figure 3:**
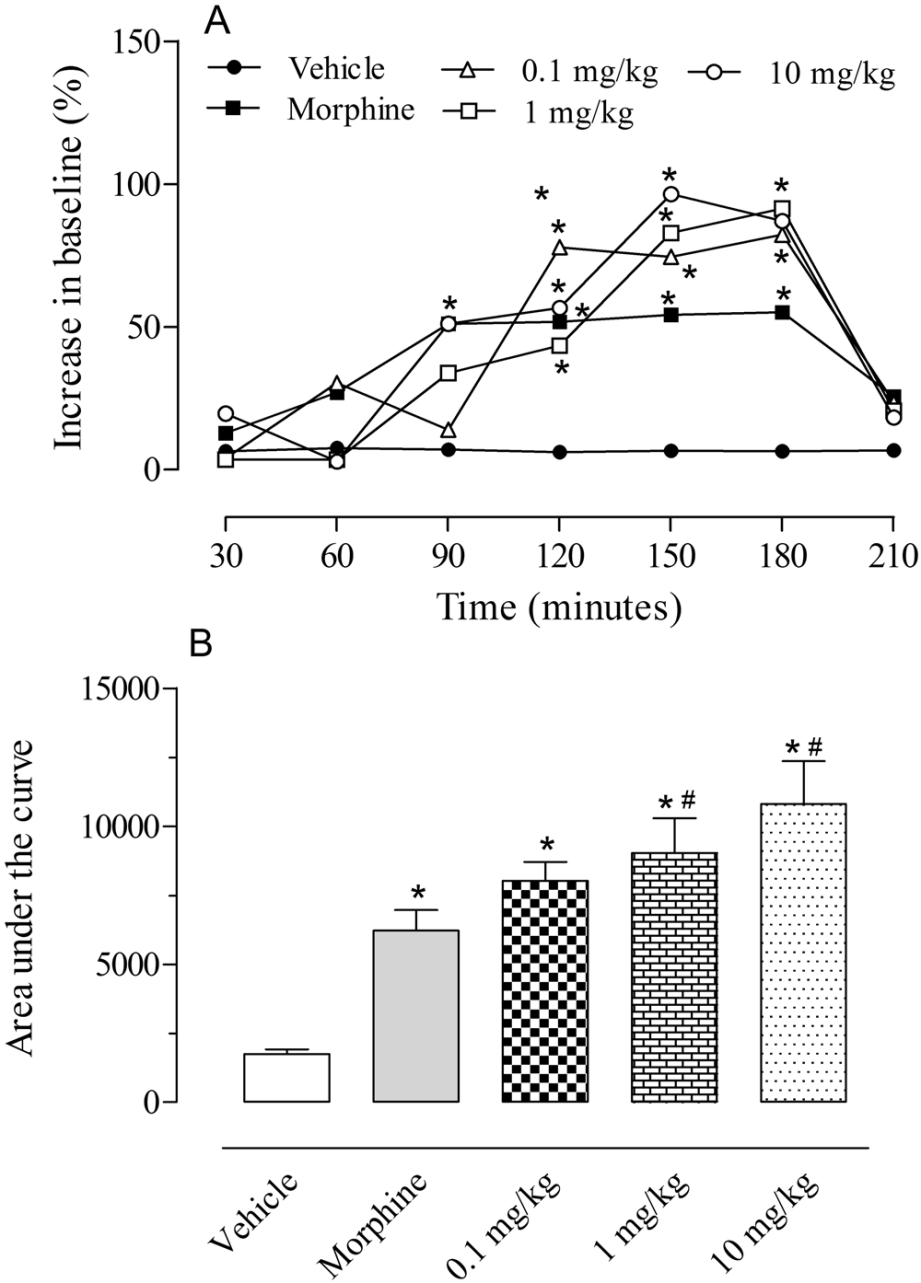
Effect of C18 5-HT on the hot plate model. Animals were orally pretreated with C18 5-HT (0.1, 1 or 10 mg/k), morphine (2.5 mg/kg) or vehicle (Tween 80). The results are presented as the mean ± S.D. (n = 6–8) of the increase in the response time relative to baseline levels (**A**) or area under the curve (**B**) calculated with the Prism Software 5.0. Two-way ANOVA followed by Bonferroni’s test was used to calculate the statistical significance. *Indicates p < 0.05 when compared to the vehicle-treated group, and ^#^Indicates p < 0.05 when compared to the morphine-treated group.

As C18 5-HT showed significant effects in the first phase of the formalin-induced licking model, we decided to evaluate the possible central antinociceptive activity of the compound. The animals were pretreated with increasing doses of C18 5-HT, and the antinociceptive effects were evaluated via the hot plate model. As shown in Fig. 3A, at 90, 120, 150 and 180 minutes after treatment with C18 5-HT (1 and 10 mg/kg), the animals showed increased antinociceptive effects from the baseline values by 33.8%, 43.4%, 82.9% and 91.5%, respectively, for the 1 mg/kg dose and 51.0%, 56.6%, 96.5% and 87.0%, respectively, for the 10 mg/kg, which were also higher than the morphine values.

When the results expressed in latency time over time were converted to an area under the curve graph, treatment with C18 5-HT at 0.1, 1 and 10 mg/kg doses showed antinociceptive and a dose-dependent profile. C18 5-HT was able to increase the AUC compared to the vehicle-treated group at the three tested doses. The 1 and 10 mg/kg doses were also able to increase the AUC compared to the morphine-treated group (Fig. 3B).

### Evaluation of the antinociceptive effects of C18 5-HT in the thermal hyperalgesia model

We further evaluated whether C18 5-HT displayed an anti-hyperalgesic effect hours after carrageenan (25 μl, 2%) intraplantar injection. The carrageenan hyperalgesic effect was observed even at 4 hours after injection and persisted up to 24 hours. A significant reduction in paw withdrawal latency (seconds) was observed 4, 6 and 24 hours after carrageenan injection, which reduced the withdrawal time from 7.0 ± 1.5 seconds (1 hour post-injection) to 3.6 ± 0.7 seconds, 4.3 ± 0.9 and 4.2 ± 1.1 seconds, respectively.

**Figure 4:**
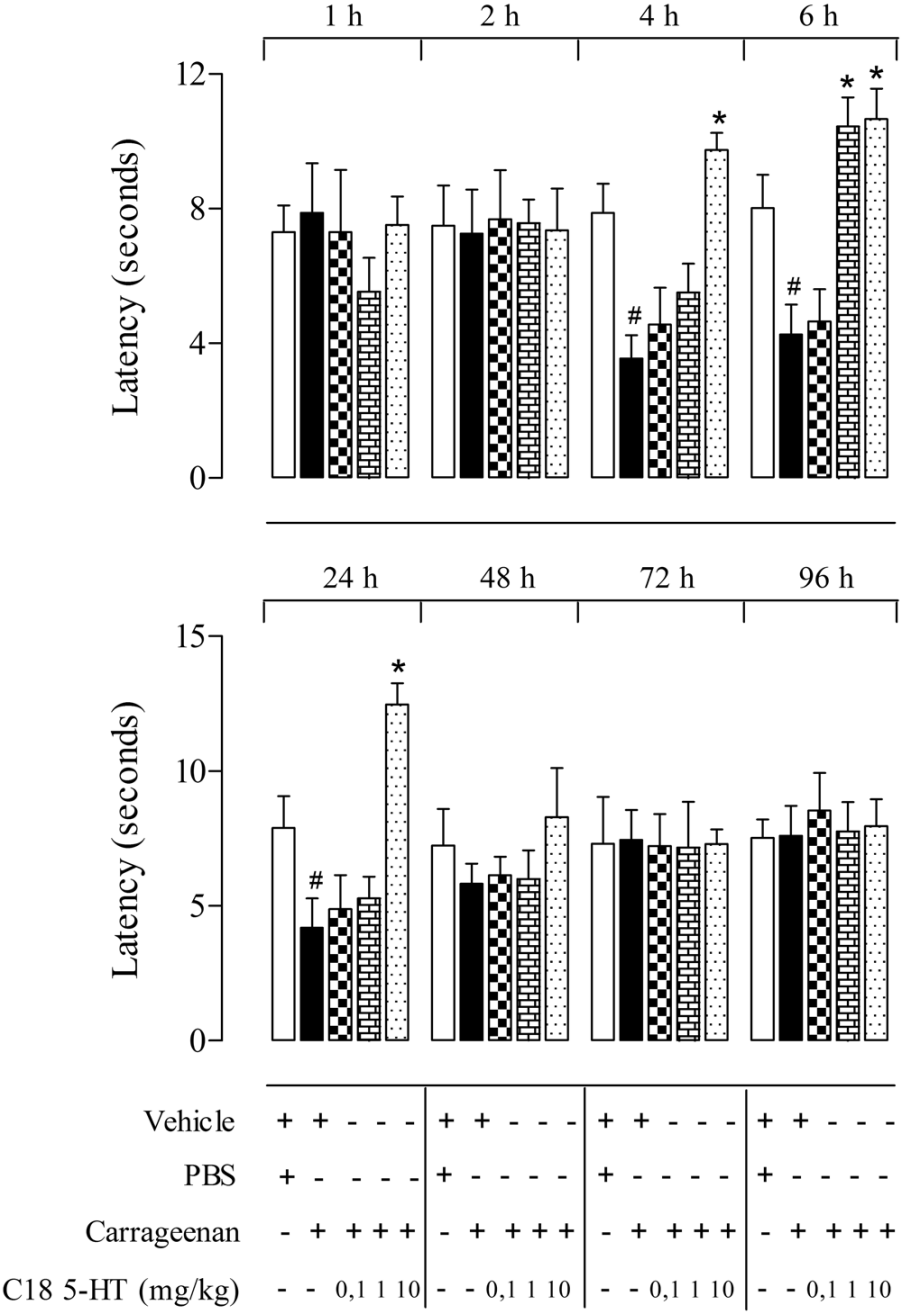
Antinociceptive effects of C18 5-HT in the thermal hyperalgesia test. Animals were pretreated orally with C18 5-HT (0, 1, 1 or 10 mg/kg) or vehicle (Tween 80) 30 min before the intraplantar injection of carrageenan (2%) and observed at 1, 2, 4, 6, 24, 48, 72 and 96 hours after this injection. The results are presented as the mean ± S.D. (n = 6–8) of the time that the animal spent licking the carrageenan-injected paw. Two-way ANOVA followed by Bonferroni’s test was used to calculate the statistical significance. *Indicates p < 0.05 when compared to the vehicle-treated mice.

Figure 4 shows that oral administration of C18 5-HT increased the paw withdrawal latency (seconds) of the animals on the hot plate 6 hours after carrageenan injection at the 1 and 10 mg/kg doses as well as 4 and 24 hours after injection at a 10 mg/kg dose, suggesting that this substance may act by inhibiting the release of inflammatory components or by increasing the excitability threshold of the nerve fibers.

### Investigation of the C18 5-HT mechanism of action

As C18 5-HT showed significant antinociceptive effects, we decided to investigate the participation of different endogenous systems involved in both nociceptive impulse transmission and in the activation of pathways that participate in the controlling nociception.

**Figure 5:**
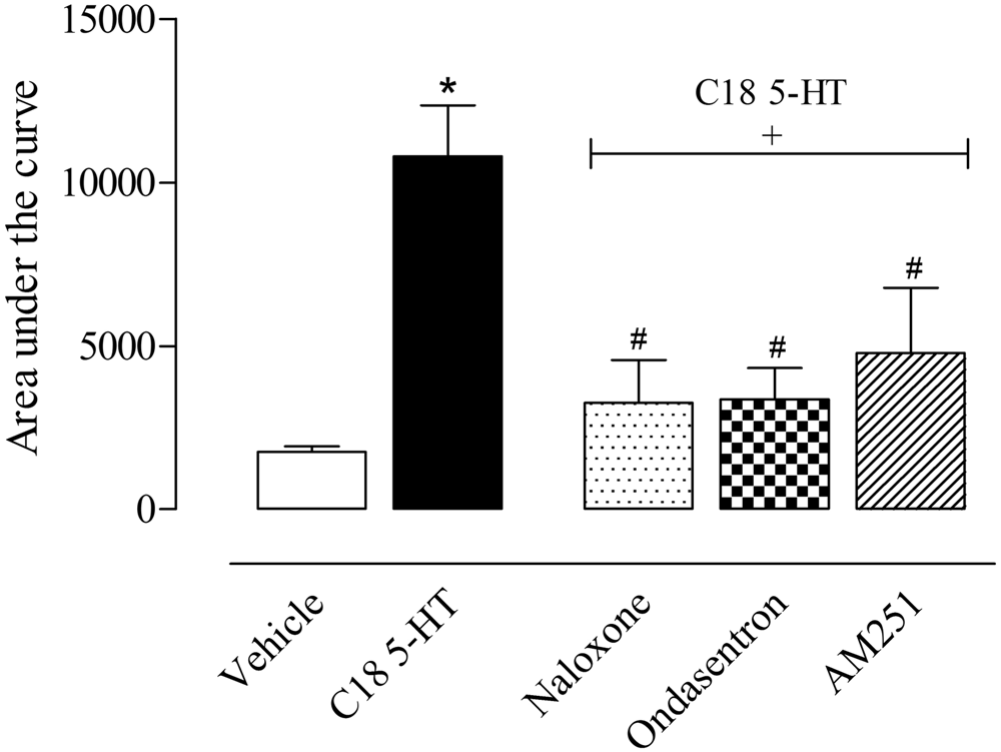
Effect of the different antagonists on the antinociceptive activity of C18 5-HT in the hot plate model. The animals were pretreated with naloxone (1 mg/kg, i.p.), ondansetron (0.5 mg/kg, i.p.) and AM251 (1 mg/kg, i.p.) 15 min before oral administration of C18 5-HT (10 mg/kg) or vehicle (Tween 80). The results are expressed as the mean ± S.D. of the percentage increase relative to the (**A**) baseline or (**B**) area under the curve calculated with the Prism Software 5.0 (n = 6–8). Two-way ANOVA followed by Bonferroni’s test was used to calculate the statistical significance. *Indicates p < 0.05 when compared to the vehicle-treated group, and ^#^Indicates p < 0.05 when comparing the antagonist-treated mice with the C18 5-HT-treated group.

The first model we used to investigate the mechanism of action was the hot plate model. Thus, the animals were pretreated with different antagonists 15 minutes before oral administration of C18 5-HT (10 mg/kg). The antagonist doses were chosen from experiments previously performed by our group and as described by Pinheiro *et al*. (2010). Pretreatment with the opioid antagonist naloxone (1 mg/kg, i.p.), the serotonergic antagonist ondansetron (0.5 mg/kg, i.p.) or the cannabinoid antagonist AM251 reversed the antinociceptive effects of C18 5-HT in the hot plate model (Fig. 5).

**Figure 6:**
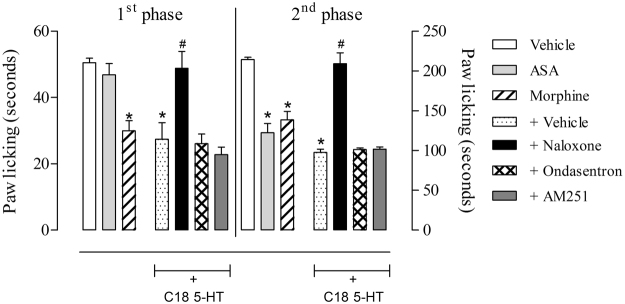
Evaluation of the participation of the opioid, serotonergic and cannabinoid systems on the antinociception effects of C18 5-HT in the formalin-induced licking model. The animals were pretreated with naloxone (1 mg/kg, i.p.), ondansetron (0.5 mg/kg, i.p.) or AM251 (1 mg/kg, i.p.) 15 minutes before oral administration of C18 5-HT (10 mg/kg). The results are expressed as the mean ± S.D. of the time the animals spent licking the formalin-injected paw (n = 6–8). Statistical significance (*p < 0.05) was calculated by analysis of variance (ANOVA) followed by the Bonferroni’s post-test between the groups pretreated with morphine, aspirin or C18 5-HT and the vehicle vehicle-treated group (*p < 0.05) or the naloxone-, ondansetron- or AM251-pretreated groups with the C18 5-HT-treated group (^#^p < 0.05).

The effects of these same antagonists were also evaluated for their capacity to reverse the C18 5-HT antinociceptive effects in the formalin-induced licking response experiment. According to the results presented in Fig. 6, the group pretreated with the vehicle displayed 50.5 ± 1.4 and 214.3 ± 3.1 second licking times in the 1^st^ and 2^nd^ phases, respectively. Naloxone was the only antagonist that reversed the antinociceptive effects of C18 5-HT in both phases (Fig. 6).

### Effect of C18 5-HT on locomotor performance, spontaneous activities and behavioral and stomach toxicity

To discard the stimulant or depressant activities and anxiolytic actions of the compound and verify whether it could promote incoordination in the animals either through sedation and/or muscle relaxation, we used the rotarod and open field models. After administration of C18 5-HT (10 mg/kg, p.o.), locomotor performance was evaluated by quantifying the number of times that each animal fell off the rotarod apparatus. The results show that the compound did not affect the number of falls compared to the results for the vehicle-treated group treated. Similarly, it was also observed that C18 5-HT did not interfering with mouse handling and exploratory capacities (Table 1). We did not observe alterations on respiration and no ulcers were observed in stomach after 5 days. Besides that, there were no alterations in normal activity, such as food and water intake, grooming, and loss of righting reflex (data not shown).

**Table 1:** Effects of C18 5-HT on mouse spontaneous activities and motor performance.

	Hour after treatment			
	0.5	1	2	3.5
*Locomotor performance*				
Vehicle	0.4 ± 0.5	0.6 ± 0.9	0.2 ± 0.4	0.2 ± 0.4
C18 5-HT	0.4 ± 0.5	0.4 ± 0.9	0.4 ± 0.5	0.2 ± 0.4
*Spontaneousactivity*				
Vehicle	54.8 ± 7.7	60.0 ± 6.6	64.2 ± 6.9	62.6 ± 4.8
C18 5-HT	62.8 ± 11.3	65.8 ± 13.7	61.4 ± 12.7	59.0 ± 6.7

Results are presented as the mean ± SD (n = 6–8).

## Discussion

The present study demonstrated, for the first time, the antinociceptive effects produced by ^β^*N*-octadecanoyl-5-hydroxytryptamide (C18 5-HT). Intraplantar administration of formalin produces nociception characterized by two distinct phases. The first phase (neurogenic phase), occurs between formalin injection and 5 minutes and is due to activation of C-fibers with activation of TRPA1 channels^8^ and reflects centrally mediated pain. The second phase (inflammatory phase), occurs between 15 and 30 minutes after formalin injection and is mediated by the release of a combination of inflammatory mediators and sensitization of central nociceptive neurons^9–11^. It is also well-known that centrally acting drugs, such as opioids (morphine and codeine), inhibit nociception in both phases, while peripheral-acting drugs, such as indomethacin and acetylsalicylic acid, inhibit only the second phase^9,12^. However, there are conflicting data in literature relating non-steroidal anti-inflammatory drugs acting in the first phase of the model. It is known that first phase is also mediated through liberation and/or synthesis of histamine and serotonine. So, such drugs that can direct or indirect act interfering with both of these pathways can also affect and reduce the first phase of formalin induced linking response^13^. C18 5-HT significantly decreased the duration of licking time at the two higher doses in both phases of pain responses in the formalin-induced licking model.

C18 5-HT also inhibited the nociceptive response caused by intraplantar injection of capsaicin and glutamate into the mouse hind paw. Capsaicin activates vanilloid receptors, such as TRPV1-type receptors, leading to the release of several neuropeptides, excitatory amino acids, nitric oxide (NO) and pro-inflammatory mediators present in the peripheral and central terminals of primary sensory neurons that contribute to the nociceptive process^14,15^. In contrast, the nociceptive response induced by glutamate is mediated by the activation of NMDA (N-methyl-D-aspartate) and non-NMDA(α-amino-3-hydroxyl-5- methyl-4-isoxazolepropionate) receptors, as well as by the release of NO, and appears to involve peripheral, spinal and supraspinal sites of action^14^. Thus, our data suggest, at least in part, that the antinociceptive effects of 5-HT C18 may be due to interactions with the glutamatergic system.

The central analgesic effect of 5-HT C18 was observed in the hot plate model. This model is considered a central reflex and is an interesting model for evaluating spinal and supra-spinal-acting drugs^16^. Animals treated with C18 5-HT demonstrated an increase in the latency time of response, demonstrating the central nociceptive activity of this compound. As several endogenous systems, such as the opioid, serotonergic and cannabinoid systems, are involved in pain control, we have investigated the mechanism of the antinociceptive actions of C18 5-HT in the hot plate and formalin**-**induced licking models. The reversal of the antinociceptive effects by an antagonist suggests the involvement of a specific inhibitory endogenous pathway. In the hot plate model, pretreatment with naloxone, ondansetron, and AM251 caused a reversion of antinociception caused by C18 5-HT, suggesting the involvement of those pathways in the antinociceptive effects of this compound.

The μ, δ, and κ opioid receptors can be found in peripheral, spinal and supraspinal regions. These receptors are densely expressed in the spinal dorsal horn (laminae I and II) in the dorsal root ganglia, the nucleus raphe magnus and the periaqueductal gray^17^. Decending pathways also involves the participation of opioid receptors, which promote a reduction in the synaptic release of γ-aminobutyric acid (GABA) from the spinal cord rostral medial projections and periaqueductal gray. The subsequent step is the promotion of spinal projections from adrenergic neurons in the *locus coeruleus*^17^. In the present study, opioid receptors were blocked with naloxone before oral administration of C18 5-HT. The antinociceptive actions of C18 5-HT were antagonized by pretreatment with naloxone by reversing its effect, which increased the latency time and area under the curve (AUC). These results suggest that the mechanism of action for C18 5-HT may involve opioid receptors.

The participation of the cannabinoid system in analgesia is due to the activation of CB1 receptors present in neurons in the dorsal root ganglion, regions of the dorsolateral funiculus and laminae I and II of the dorsal horn in the spinal cord. Several studies have indicated that CB1 receptor activation exerts antinociception against stimuli such as capsaicin and formalin^17–19^. Our results showed that the cannabinoid antagonist AM251 (1 mg/kg, i.p) reversed the antinociceptive effects of C18 5-HT in the hot plate model, suggesting that the cannabinoid system may be involved our compound’s mechanism of action. It is noteworthy that cannabinoids and opioids share common sites of action in the central nervous system, as each produces antinociception when given intrathecally^20^ or intracerebroventricularly^21^. Moreover, both classes of drugs appear to produce antinociception through the activation of descending monoaminergic systems^22^.

Involvement of the serotonergic system in the antinociceptive effect was observed in C18 5-HT administration after pretreatment with the 5-HT_3_ receptor antagonist ondansetron. This antagonist was able to reverse the effects of C18 5-HT in the hot plate model, suggesting that this system may be involved in the mechanism of action of our compound. It is known that serotonin can act through several receptors to inhibit or facilitate nociceptive transmission^23^. The reversal of the C18 5-HT antinociceptive effects by ondansetron indicates that the serotoninergic pathway contributes, at least in part, to the action of this substance. This action could be related to the production and release of serotonin and/or a direct antagonistic action at 5-HT_3_ receptors located at primarily afferent fiber ends. In the formalin**-**induced licking model, only naloxone reversed the antinociceptive effects of C18 5-HT. This work showed that pretreatment with naloxone blocked the antinociception produced by C18 5-HT in formalin-induced paw licking, indicating the involvement of peripheral opioid receptors in antinociceptive action by C18 5-HT during systemic administration.

Hyperalgesia is characterized by inflammatory pain that involves both the sensitization of peripheral nociceptors and the central transmission of the dorsal horn of the spinal cord to the thalamus where the pain is perceived^24^.Carrageenan-induced inflammation was originally described by Winter *et al*.^25^ as an acute, highly reproducible and non-immune response. Cardinal signs of inflammation, such as edema, hyperalgesia, pain and erythema, develop after the subcutaneous injection of carrageenan, which result from the actions of pro-inflammatory agents such as bradykinin, histamine, eicosanoids, and oxygen free radicals released directly into the animal’s paw. These agents may be generated at the injury site or by migrating cells. According to Lavich *et al*.^26^, plantar stimulation in rats with carrageenan (50 μg/paw) leads to a rapid hyperalgesic response in the injected paw that can be evaluated by the thermal hyperalgesia test. In our study, we found that after intraplantar injection of carrageenan mice reacted to the thermal plantar stimulation with an acute decrease in the latency of the withdrawal response of the ipsilateral paw in comparison to paws injected with the vehicle. Our findings indicated that the paw withdrawal latency created by carrageenan decreased by approximately 8 to 4 seconds compared with the paw withdrawal latency of the vehicle-treated paw at 4, 6 and 24 h after carrageenan administration. This indicates that the carrageenan challenge was capable of triggering a hyperalgesic response, which was very rapid in onset, peaked within 4 to 6 h post-challenge and remained significant for at least 24 h. Oral administration of C18 5-HT was able to decrease the hyperalgesic effect induced by carrageenan in mice, which increased the latency time of the animals at 4, 6 or 24 hours after carrageenan injection. These results are similar to those shown by Lavich *et al*.^26^, where treatment with indomethacin (4 mg/kg, i.p.) 1 h before challenge prevented the carrageenan-evoked hyperalgesic response from 15 to 360 minutes. Thus, these results suggest that C18 5-HT has anti-hyperalgesic effect.

To confirm the antinociceptive effects of C18 5-HT, we removed the stimulant or depressant activities and even the anxiolytic actions in the animals pretreated with the compounds and evaluated the effects using the rotarod and open field models. Our results showed that C18 5-HT did not affect either the rotarod or open field models, indicating that the effects observed in the other models are due to the antinociceptive effects and not influenced by motor impairments or sedation.

Comparing the molecules of serotonin and C18 5-HT the only similarity between both of them is that the molecule of serotonin was used in the synthesis of the new substance. Serotonin have a free amino (NH2) group and C18 5-HT presents an acyl chain. This amidic bond is very strong and similar to a peptidic bond. Chemically, amide and amine are functional clusters with distinct bonds made at the receptors. In addition, the amide has a long chain, which changes the conformation of the molecule and its spatial configuration, causing different interactions with the receptors to occur. In this regard, a large carbon chain (with 18 atoms) was added originating C18 5-HT. The new molecule do not activate the serotonin receptors probably due to its long chain, high molecular weight and/or spatial configuration.

## Conclusions

Taken together, the results show the first evidence for the antinociceptive effects of C18 5-HT. Its mechanism of action appears to involve, at least in part, the activation of the opioid, serotoninergic and cannabinoid systems and has anti-hyperalgesic effects, as observed in the thermal hyperalgesia model. According to these results, this compound may be a novel prototype for future analgesic drugs.

## Material and Methods

### Animals

Male and female Swiss *Webster* mice (20–25 g, 8–10 weeks) donated by the Instituto Vital Brazil (Niterói, Rio de Janeiro, Brazil) were used in this study. Animals were housed in a temperature-controlled room at 22 ± 2 °C with a cycle of 12 h light/dark and free access to food (Nutrilab, Brazil) and water. Before each experiment (12 hours) mice received only water in order to avoid food interference with substances absorption. Mice were daily monitored to physical condition and if any signs of suffering was observed, animal was euthanized. The research was conducted in accordance with the internationally accepted principles for laboratory animal use and care. The experimental protocols used in this work followed the rules advocated by Law 11,794, of October 8, 2008 by the National Council of Animal Experimentation Control (CONCEA) and were approved by the Ethics Committee of Animal Use (CEUA), Science Center Health/UFRJ and received the number DFBCICB015-04/16.

### Drugs and treatment

The following drugs were used: Acetylsalicylic acid (ASA), AM251 (*N*-(piperidin-1-yl) (4-iodophenyl)-1-(2,4-dichlorophenyl)-4-methyl-1*H*-pyrazole-3-carboxamide), capsaicin, L-glutamic acid (glutamate), capsazepine and ondansetron (Sigma-Aldrich, St. Louis, MO, USA); Morphine sulfate and naloxone hydrochloride kindly provided by Cristália (São Paulo, Brazil); formalin (Isofar (Rio de Janeiro, Brazil); C18 5-HT (synthesized in our laboratories). C18 5-HT was dissolved in dimethylsulfoxide (DMSO) to prepare a 100 mg/ml stock solution. This solution was administered by oral gavage at doses varying between 0.1 and 10 mg/kg in a final volume of 0.1 ml of Tween 80 per animal. In all the experiments, the final concentration of DMSO or Tween 80 had no effects *per se*. All drugs were diluted just before use and administered orally (p.o.), intraperitoneally (i.p.) or via an intraplantar route (i.pl.). Doses were choosed based on preliminary experiments made in our laboratory (Pinheiro *et al*., 2010), where the results of doses above 10 mg/kg were statistically equal to 10 mg/kg.

### C18 5-HT synthesis

**Figure 7:**
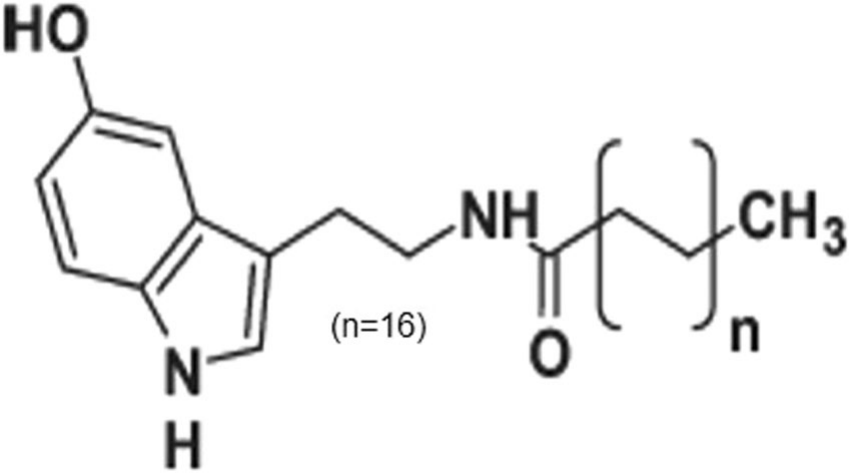
Structure of ^β^*N*-octadecanoyl-5-hydroxytryptamide (C18 5-HT).

Stearoylchloride (1.01 mmol) was added drop wise to a mixture of serotonin hydrochloride (1 mmol) and NaHCO_3_ (3 mmol) in 3 ml of THF with 1 mL of distilled H_2_O under a nitrogen atmosphere and stirred for 4 h at room temperature. Next, the mixture was diluted in 250 ml dichloromethane, and the organic phase was washed with 300 ml of water (3 × 100 mL), dried under Na_2_SO_4_ and filtered. The solvent was evaporated, and the residue was resuspended in 1 ml of THF before 40 mL of petroleum ether was added drop wise. The solution was then centrifuged to give a white solid with a 74% yield^26^. The final structure was confirmed by RMN (Fig. 7)

### Formalin-induced licking model

The mouse licking model is characterized by a biphasic response: The first phase is an acute neurogenic pain response of short duration that occurs during the first 5 min after the intraplantar injection of formalin (2.5%). The second longer-lasting tonic phase occurs between 15 and 30 min post injection and is an inflammatory pain response. Animals were given 20 μl of formalin (2.5% v/v) into the dorsal surface of the left hind paw. The licking time of the formalin-injected paw was immediately recorded during these two phases^9,27^. Animals were orally pretreated with differents doses of the C18 5-HT (0.1–10 mg/kg), morphine (2.5 mg/kg), ASA (200 mg/kg), or vehicle (in Tween 80) 60 min before the formalin intraplantar administration.Capsaicin-induced licking model

This protocol was described by Sakurada *et al*.^28^ and adapted by Giorno *et al*.^29^. An intraplantar injection of capsaicin (20 μl, 1.6 μg/paw) was administered to the right hind paw 60 minutes after oral administration of C18 5-HT (0.1–10 mg/kg) or vehicle. Immediately, the animals were individually placed in a transparent box, and the time that the animal spent licking or biting the capsaicin-injected paw was recorded over a period of 5 minutes.

### Glutamate-induced licking model

The glutamate induced licking model in mice was described by Beirith *et al*.^13^ and adapted by Giorno *et al*.^29^. Mice were given C18 5-HT p.o. (0.1–10 mg/kg) or vehicle 60 min before the intraplantar injection of glutamate (20 μl, 3.7 ng/paw) and were individually placed in a transparent box; the number of licking and bitng behevior was then counted for 15 minutes.

### Hot Plate Model

At intervals of 30 min after oral administration of C18 5-HT (0.1–10 mg/kg), vehicle or morphine, animals were placed on a hot plate (Insight Equipment, Brazil) set at 55 ± 1 °C. The reaction time was recorded when the animals licked their fore- and hind-paws. At 60 and 30 min before C18 5-HT administration, the vehicle or morphine mean of two reaction time measurements was calculated, and this value was considered the baseline. The experimental model was first described by Woolfe and Macdonald^30^ and was adapted by Matheus *et al*.^31^. Antinociception was quantified as the area under the curve (AUC) for responses from 30 min after drug administration until the end of the experiment. The following formula, which is based on the trapezoid rule, was used to calculate the AUC: AUC = 30 × IB [(min 30) + (min 60) + … + (min 180)/2], where IB is the increase from the baseline (in %).

### Analysis of the Mechanisms of Action of C18 5-HT

To assess the possible mechanism of the antinociceptive actions of C18 5-HT, we investigated the participation of the opioid, serotonergic and cannabinoid systems. One of the following treatments was given intraperitoneally (i.p.) 15 min before C18 5-HT (10 mg/kg, p.o.): (1) naloxone (opioid receptor antagonist, 1 mg/kg, i.p.), (2) ondansetron (serotonergic receptor antagonist, 0.5 mg/kg, i.p.) or (3) AM251 (cannabinoid receptor antagonist, 1 mg/kg, i.p.).

The antagonist doses were based on the literature^32^. Dose response curves for each antagonist against the respective agonist were previously constructed, and the dose that reduced the agonist effect by 50% was chosen for the assays. The antinociceptive effects were evaluated via the hot plate test and in the formalin-induced licking model as described above.

### Thermal Hyperalgesia Model

Thermal hyperalgesia was induced by carrageenan (2%, 25 µl/paw, i.pl.) according to the method described by Sammons *et al*.^33^. Animals received carrageenan in the right hind paw 30 minutes after oral administration of C18 5-HT (0.1–10 mg/kg) or vehicle and then were placed on a hot plate (Insight Equipment, Brazil) set at 55 ± 1 °C at 1 h intervals (up to 96 h after the treatment). The time (in seconds) necessary for the animals to jump and/or lick the injected paw was recorded with the aid of a stopwatch and named the “paw withdrawal latency”. Reductions in response times were viewed as indicative of the development of inflammatory hyperalgesia.

### Locomotor Performance Evaluation and Spontaneous Activity

To evaluate the effects of C18 5-HT on locomotor performance, we used the rotarod apparatus^34–36^. Twenty-four hours before the experiments, all the animals were trained until they could remain on the device for 60 seconds without falling. On the day of the experiment, the mice were treated with vehicle or C18 5-HT (10 mg/kg, p.o.) and tested on the Rota Rod 0.5 h to 3.5 h after administration. The number of falls by the mice was recorded up to 240 seconds.

The spontaneous activity of C18 5-HT was evaluated by the Open Field Test. This test was based on the methodology adapted by Figueiredo *et al*.^37^. Mice were orally pretreated with C18 5-HT (10 mg/kg) or vehicle and individually placed in an observation chamber where the floor was divided into 50 squares (5 cm × 5 cm) 60 minutes after treatment. The exploratory activity of the animals was evaluated over 5 minutes, and the total number of squares traversed by the animals was counted.

### Behavioral and stomach observations

Signs of acute toxicity, such as behavioral parameters (i.e. convulsion, hyperactivity, sedation, grooming, loss of righting reflexes, or increased or decreased respiration), as well as food and water intake, were observed over a 5 day period after a single oral dose of C18 5-HT (100 mg/kg) was administered to a group of ten animals of both sexes^38^. After this period, the animals were sacrificed by ketamine/xylazine overdose, and their stomachs were removed. An incision was made along the great curvature, and the presence of ulcers or perforations and degree of hyperemia was observed and counted.

### Data analysis

Each experimental group consisted of 6 to 8 mice, and the results are expressed as the mean ± S.D. The area under the curve (AUC) was calculated using the Prism Software 5.0 (GraphPad Software, La Jolla, CA, USA). Significant differences between the groups were established using Bonferroni’s test for multiple comparisons after two-way analysis of variance (ANOVA) testing. P values less than 0.05 were considered significant.

## References

[1] Meeks NM, Glass JS, Carroll BT (2015). Acute pain management in dermatology - Mechanisms and pathways. J. Am. Acad. Dermatol..

[2] Argoff CE (2014). Recent management advances in acute postoperative pain. Pain Pract..

[3] Maione S (2007). Analgesic actions of N-arachidonoyl-serotonin, a fatty acid amide hydrolase inhibitor with antagonistic activity at vanilloid TRPV1 receptors. Br. J. Pharmacol..

[4] Speer K, Kölling-Speer I (2006). The lipid fraction of the coffee bean. Braz. J. Plant. Physiol..

[5] Lang R, Hofman T (2005). A versatile method for the quantitative determination of b N -alkanoyl-5-hydroxytryptamides in roasted coffee. Eur. Food. Res. Technol..

[6] Meijerink J, Balvers M, Witkamp R (2013). N-acyl amines of docosahexaenoic acid and other n−3 polyunsatured fatty acids – from fishy endocannabinoids to potential leads. Br. J. Pharmacol..

[7] Ortar G (2007). New *N*-Arachidonoyl serotonin analogues with potential “dual” mechanism of action against pain. J. Med. Chem..

[8] McNamara. CR (2007). TRPA1 mediates formalin induced pain. Proc. Natl. Acad. Sci. USA.

[9] Hunskaar S, Hole K (1987). The formalin test in mice: dissociation between inflammatory and non-inflammatory pain. Pain.

[10] Omote. K (1998). Formalin induced release of excitatory amino acids in the skin of the rat hindpaw. Brain Res..

[11] Tjolsen A (1992). The formalin test: an evaluation of the method. Pain.

[12] Shibata M (1989). Modified formalin test: characteristic biphasic pain response. Pain.

[13] Parada CA, Tambeli CH, Cunha FQ, Ferreira SH (2001). The major role of peripheral release of histamine and 5-hydroxytryptamine in formalin-induced nociception. Neuroscience.

[14] Beirith A, Santos ARS, Calixto JB (2002). Mechanisms underlying the nociception andpawoedema caused by injection of glutamate into the mouse paw. Brain Res..

[15] Sakurada T, Komatsu T, Sakurada S (2005). Mechanisms of nociception evoked by intrathecal high-dose morphine. Neurotoxicology.

[16] Matsumotoa K (2004). Antinociceptive effect of 7-hydroxymitragynine in mice: Discovery of an orally active opioid analgesic from the Thai medicinal herb *Mitragyna speciosa*. Life Sciences.

[17] Pan HL (2008). Modulation of pain transmission by G-protein-coupled receptors. Pharmacol. Ther..

[18] Hohmann AG (2004). Selective activation of cannabinoid CB2 receptors suppresses hiperalgesia evoked by intradermal capsaicin. J. Pharmacol. Exp. Ther..

[19] Rea K, Roche M, Finn DP (2007). Supraspinal modulation of pain by cannabinoids: the role of GABA and glutamate. Br. J. Pharmacol..

[20] Lichtman AH, Martin BR (1991). Spinal and supraspinal components of cannabinoid-induced antinociception. J. Pharmacol. Exp. Ther..

[21] Martin WJ (1993). Antinociceptive actions of cannabinoids following intraventricular administration in rats. Brain Res..

[22] Lichtman AH, Martin BR (1991). Cannabinoid induced antinociception is mediated by a spinal a2 noradrenergic mechanism. Brain Res..

[23] Kayser V (2007). Mechanical thermal and formalin-induced nociception is differentially altered in 5-HT_1A_−/−, 5-HT_2A_−/−, 5-HT_3A_−/− and 5-HT−/− knockout male mice. Pain.

[24] Menéndez L (2002). Unilateral hot plate test: a simple and sensitive method for detecting central and peripheral hyperalgesia in mice. J. Neurosc. Methods..

[25] Winter CA, Risley EA, Nuss GW (1962). Carrageenin-induced edema in hind paw of the rat as an assay for antiiflammatory drugs. Proc. Soc. Exp. Biol. Med..

[26] Lavich TR (2005). A novel hot-plate test sensitive to hyperalgesic stimuli and non-opioid analgesics. Braz. J. Med. Biol. Res..

[27] Matsui T (2002). Highly potent inhibitors of TNF-alpha production. Part II: metabolic stabilization of a new found chemical lead and conformation al analysis of an active diastereoisomer. Bioorg. Med. Chem..

[28] Gomes NM (2007). Antinociceptive activity of amazonian Copaiba oils. J. Ethnopharmacol..

[29] Sakurada T (1998). Involvement of spinal NMDA receptors in capsaicin-induced nociception. Pharmacol. Biochem. Behav..

[30] Giorno, T. B. S. *et al*. Antinociceptive effect and mechanism of action of isatin, N-methyl isatin and oxopropyl isatin in mice. *Life Sciences*, 1–38 (2013). 10.1016/j.lfs.2016.02.052 26883976

[31] Woolfe G, Macdonald AD (1944). The evaluation of the analgesic action of pethidine hydrochloride (demerol). J. Pharmacol. Exp. Ther..

[32] Matheus ME (2005). Evaluation of the antinociceptive properties from *Brillantaisia palisotii* Lindau stems extracts. J. Ethnopharmacol..

[33] Pinheiro MMG (2010). Antinociceptive activity of fractions from *Couroupita guianensis* Aubl. leaves. J. Ethnopharmacol..

[34] Sammons MJ (2000). Carrageenan-induced thermal hyperalgesia in the mouse: role of nervegrowth factor and the mitogen-activated protein kinase pathway. Brain Res..

[35] Dunham NW, Miya TS (1957). A note on a simple apparatus for detecting neurological deficit in rats and mice. J. Am. Pharmaceut. Assoc..

[36] Godoy MCM (2004). α2 Adrenoceptors and 5-HT receptors mediate the antinociceptive effect of new pyrazoles, but not of dipyrone. Eur. J. Pharmacol..

[37] Figueiredo GSM (2013). Convolutamydine A and synthetic analogs have antinociceptive properties in mice. Pharmacol. Biochem. Behav..

[38] Lorke D (1983). A new approach to practical acute toxicity testing. Arch. Toxicol..

